# Retrieval of a dislodged porth catheter with inaccessible tips in a pediatric cancer patient

**DOI:** 10.1186/s42155-021-00241-7

**Published:** 2021-06-15

**Authors:** Murat Dökdök

**Affiliations:** Anadolu Medical Center Hospital affilliated with John’s Hopkin, Cumhuriyet M. 2255 S. No 3, 41400 Gebze, Kocaeli Turkey

To the Editor,

We read the article entitled “Pigtail through snare technique: an easy and fast way to retrieve a catheter fragment with inaccessible ends” with great interest. Here, a modified technique, hooking the catheter fragment with a pigtail catheter advanced through the snare loop, is described by the authors to retrieve the catheter fragment with inaccessible ends in a prompt fashion (Mori et al. 2021). Dislodged venous catheter fragments are retrieved successfully by interventional radiologists for decades using mainly two methods: loop snare or grasping forceps. Besides, other modified adjunct methods with catheters and wires have also been described in the literature. To our best knowledge, using a pigtail catheter positioned to the optimal site when there were no free ends of the catheter was first described by Cheng et al. (2009). They also had utilized basket successfully in patients when tips of the fragments were inaccessible.

There are some other single-center experiences, including relatively small case series in the literature; most of them focus on the materials and complications; none but one reports fluoroscopy time and radiation doses (Kalińczuk et al. 2016). In this study, the median fluoroscopic time reported is 6.1 min (0.47–42.0), while the maximum dose in the longest procedure entrance skin exposure is 912 mGy. However, no specific data was given related to the catheter fragment tip accessibility. We just recently retrieved a dislodged venous port catheter with inaccessible tips in an 8-year old girl who has been treated with medulloblastoma. Venography revealed an embedded proximal catheter tip in the right internal jugular vein wall and an inaccessible distal catheter tip in the right ventricle wall without any free movements. We used a 5 Fr pigtail catheter and 0.035″ wire to hook up the central portion of the port catheter, similar to the authors. Then the silicon port catheter was withdrawn from inferior vena cava with 20 cm Amplatz GooseNeck Snare (Medtronic, USA) through the 6F catheter lumen readily. The fluoroscopy time (57 min), air kerma was kept relatively low (734 mGy) using collimators. Compared to radiation doses above study (Kalińczuk et al. 2016), it is relatively low yet still higher than the authors’ patient skin dose of 68.12 mGy (Mori et al. 2021) due to longer procedure time.

We think that time and radiation dose should be a major concern, especially when dealing with fragile pediatric patients. Advanced search in PubMed reveals a few case reports in neonates and only two case reports in pediatric cancer patients with venous catheter migration (Elgehiny et al. 2020; Eryilmaz et al. 2012). Neither duration of the procedure nor radiation doses were emphasized in these reports. Since no guideline exists about the dose concerns during such procedures, the principle of ‘ALARA’ should be implemented in all cases. Although the success of such procedures is quite high, it would be better not to use the trial and error method by trying many different materials which increases the procedure time. After diagnosing an embedded or inaccessible catheter tip, the best strategy should be executed using the appropriate technique.

## Data Availability

The datasets used during the current study are available from the corresponding author on reasonable request.
